# Impact of Fixed-Dose Combination Versus Single-Component Therapy for Benign Prostatic Hyperplasia-Related Urinary Symptoms on Persistence, Adherence, and Satisfaction in a Real-Life Setting

**DOI:** 10.3390/ph18101439

**Published:** 2025-09-25

**Authors:** Mateusz Małkowski, Anna Chudek, Agnieszka Almgren-Rachtan, Jerzy Tadeusz Chudek, Piotr Ludwik Chłosta

**Affiliations:** 1 Adamed Pharma S.A., 05-152 Czosnow, Poland; malkowski.m@proton.me; 2 Department of Pharmacovigilance, Europharma Research & Science Centre Co., Ltd., 40-061 Katowice, Poland; anna.m.chudek@gmail.com; 3 Health Promotion and Obesity Management Unit, Department of Pathophysiology, Faculty of Medical Sciences in Katowice, Medical University of Silesia in Katowice, 40-752 Katowice, Poland; 4 Department of Internal Medicine and Oncological Chemotherapy, Medical Faculty in Katowice, Medical University of Silesia, 40-055 Katowice, Poland; 5 Department of Urology, Jagiellonian University Medical College, 31-008 Krakow, Poland; piotr.chlosta@uj.edu.pl

**Keywords:** lower urinary tract symptoms, benign prostatic hyperplasia, fixed-dose drugs, real-life data, everyday clinical practice, persistence, adherence

## Abstract

**Background:** Fixed-dose combination medications (FDCs) are recognized methods of increasing adherence to polytherapy in chronic diseases. However, the role of FDCs in patients with benign prostatic hyperplasia (BPH) associated with lower urinary tract symptoms (LUTS) remains uncertain. We designed this study to assess persistence, adherence, and patient satisfaction with FDCs recently introduced to the Polish pharmaceutical market, which contain tamsulosin (an α1-adrenergic receptor antagonist) in combination with solifenacin (a muscarinic receptor antagonist) or dutasteride (a 5-α reductase inhibitor). **Methods:** The analysis included 50,435 men (67.8 ± 8.8 years old) managed by urologists for BPH-associated LUTS, who had been on combination therapies for at least 3 months. Two study visits, with an interval of 2.1 ± 1.4 months, were conducted between February and December 2024. **Results:** Single-component drugs (83.1%) were more common forms of therapy compared to FDCs (16.9%). ARAs (α1-adrenergic receptor antagonists) with 5-α reductase inhibitors comprised 70.2%, while ARAs with muscarinic receptor antagonists or β3-adrenergic agonists comprised 29.5%. Persistence with therapy across two visits was 82.0% for single-component drugs and 93.6% for FDCs (*p* < 0.001); OR = 1.31 (95% CI: 1.02–1.63). Similarly, adherence was better in patients treated with FDCs (96.6% vs. 91.0% at visit 1, *p* < 0.001; 99.3% vs. 97.9% at visit 2, *p* < 0.05). Patients prescribed FDCs were satisfied with therapy more often than those prescribed single-component drugs (62.6% and 76.8% vs. 50.6% and 67.5% at visits 1 and 2, respectively; *p* < 0.001). **Conclusions:** 1. Combination therapies are still more commonly administered as separate tablets than FDCs in patients with BPH-associated LUTS. 2. The use of FDCs increases short-term satisfaction and persistence with therapy, with a mild effect on adherence.

## 1. Introduction

According to the recommendations of the European Association of Urology (EAU), pharmacological treatment of non-neurogenic lower urinary tract symptoms (LUTS) accompanying benign prostate hyperplasia (BPH) includes the use of α1-adrenergic receptor antagonists (ARAs) and 5-α reductase inhibitors (5-αRIs); muscarinic receptor antagonists (MRAs); mirabegron, the only licensed β3-agonist, tadalafil from phosphodiesterase type 5 inhibitors; and phytotherapy [1]. Pharmacological treatment should be initiated in patients with moderate-to-severe symptoms (≥8 points), as assessed based on the IPSS (International Prostate Symptom Score), which is equivalent to the AUASI (American Urological Association Symptom Index). The myorelaxant effect of ARAs and their action on prostate and bladder neck stromal cells appear shortly after therapy initiation. Still, these drugs do not prevent acute urinary retention (AUR) or the need for surgery. In particular, 5-αRIs are recommended in patients with prostate enlargement >40 mL in combination with ARAs. By inhibiting the conversion of testosterone and androstenedione to dihydrotestosterone, 5-αRIs induce prostatic cell apoptosis, resulting in a decrease in prostate volume and reducing the risk of AUR by 57% and the risk of surgical intervention (TURP—Transurethral Resection of the Prostate) by 55% [2]. They act slowly over months of treatment. Combining drugs from the abovementioned groups results in more effective inhibition of disease progression, reducing the risk of AUR by 68% and BPH-related surgery by 71% [3]. Adding MRA to ARA can alleviate symptoms related to disturbed bladder filling and urine storage more effectively, reducing urinary urgency, urge urinary incontinence, voiding frequency, and nocturia [4].

Therefore, the current standard of treatment for symptoms of overactive bladder (OAB) accompanying BPH includes the use of MRAs or the β3-adrenergic agonist [5,6]. The drugs mentioned above increase bladder capacity by relaxing the detrusor muscle and inhibiting its uncontrolled contraction without expanding residual urine volume after urination. They may even exhibit some local analgesic effects. Their use reduces the frequency of urinary urgency and extends the time to the sensation of urgency or the need to urinate, thereby improving quality of life by reducing restrictions on partner, family, social, and professional activities [7,8].

Combined therapy (polytherapy) is increasingly used to treat BPH-associated LUTS [9]. Fixed-dose combination pills (FDCs) are a well-recognized method that improves adherence and persistence to polytherapy in chronic diseases, especially hypertension [10]. However, the improvement of adherence and persistence with FDCs in men with BPH-associated LUTS is not consistent in international studies. According to German prescription database (IQVIA), persistence with an FDC of tamsulosin and dutasteride in men with BPH was only slightly greater after 12 months (41.8% vs. 41.0%) and 24 months (28.2% and 27.1%), but adherence (measured by the medication possession ratio) was significantly better compared to a group using two separate single-component medications [11]. Much greater differences were described in other populations. In the Netherlands, an analysis of the prescription records database revealed that FDCs containing tamsulosin and solifenacin extended median persistence from 112 to 414 days [12]. At 12 months, 51.3% of patients continued treatment with FDCs, compared to 29.9% on therapy based on separate products. Similarly, a study conducted in the Spanish population, utilizing the Spanish IQVIA Cegedim Electronic Medical Records database, showed that the median time to discontinuation of treatment in men with LUTS using FDCs (84.4% tamsulosin with solifenacin) was 125 days. In contrast, the use of two separate tablets was associated with a median time to discontinuation of therapy of only 31 days [13]. An analysis of persistence in an Italian prescription administrative database included patients with BPH-associated LUTS exposed to at least 6 months of therapy [14]. They showed a 1-year persistence with FDCs of only 9%, with a long-term 5-year persistence of 3%. In the group treated with FDCs, 31.1% of patients continued treatment for 12 months, whereas in the group using separate tablets, the rate was only 8.9%. The use of FDCs was associated with three times less frequent discontinuation of treatment [14].

A large survey conducted in 2012–2013 among outpatients with BPH in Poland revealed that monotherapy with ARAs was used with comparable frequency to polytherapy with ARAs and 5-αRI (47.5% and 47.9%, respectively) [15]. Until now, there has been a lack of data concerning the use of FDCs in the management of BPH-associated LUTS in Poland.

The appearance of FDCs containing tamsulosin with dutasteride or solifenacin on the Polish pharmaceutical market prompted us to conduct this study, aiming to assess their implementation in patients with BPH-associated LUTS. In addition, we studied persistence, adherence, and patient satisfaction.

## 2. Results

### 2.1. Study Group Characteristics

The analysis included 50,435 patients with BPH-associated LUTS, with a mean age of 67.8 ± 8.8 years, who were treated with combination therapies. The patients’ level of education varied, with a small percentage of men having completed primary education. Professionally inactive men constituted 69.9% of the study group, and only 16.0% lived in rural areas (Table 1).

Moderate-to-severe BPH-associated LUTS were reported by 86.2% of participants, and these symptoms significantly worsened the quality of life of 14.6% of patients. Significant prostate enlargement (>40 mL) was reported for 41.2% of men, and a PSA level >20 ng/mL was found in 0.2%.

The most frequently reported symptoms of OAB included urinary urgency, nocturia, and daytime frequency. Urinary incontinence was a relatively less common symptom. As many as 17.4% of the study participants reported experiencing four or more symptoms. Neurological diseases that may cause an overactive bladder occurred in 4.6% of the study participants (Table 1).

Comorbidity, including cardiovascular diseases, diabetes, and recurrent urinary tract infections, was reported in 92.0% of the study participants (Table 1).

### 2.2. Pharmacotherapy

**Figure 1:**
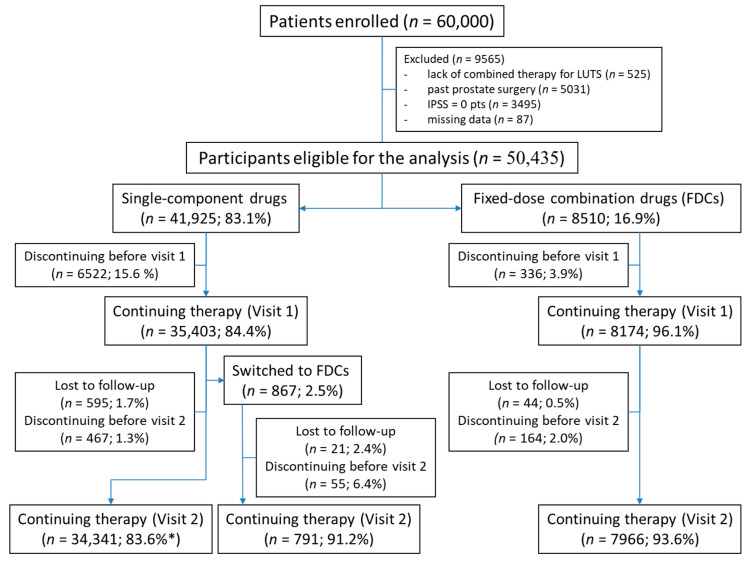
Analysis flowchart. * Percentage calculated for patients who did not switch to fixed-dose combination drugs at visit 1.

Regarding forms of combined therapy, single-component drugs (83.1%) were used more commonly than FDCs (16.9%); see Figure 1. The most frequently used combined therapy was ARA with 5αRI, followed by ARA with MRA or β3-adrenergic agonists (70.2 vs. 29.5%, *p* < 0.001). FDCs containing ARA with solifenacin were prescribed more frequently than FDCs of ARA with 5αRI (71.0% vs. 29.0%). The medication regimen remained unchanged for at least one year in 53.1% of patients on single-component preparations and in 72.5% of patients on fixed-dose combinations (FDCs) (*p* < 0.001); see Table 2.

The most frequently used ARA and 5αRI combination therapies administered as single-component drugs were tamsulosin with finasteride (58.1%), tamsulosin with dutasteride (11.5%), doxazosin with finasteride (6.8%), and doxazosin with dutasteride (2.2%). Silodosin was prescribed sporadically, while terazosin was not used. The most frequently used single-component preparations not available as FDCs on the Polish market included finasteride and doxazosin.

Standard combination therapies of ARA with solifenacin (without 5α-RIs) included single-component drugs, such as tamsulosin (15.8%) or doxazosin (3.3%). Mirabegron was rarely used. Tamsulosin with solifenacin FDC was used in 40.6% of subjects receiving any ARA with solifenacin in all analyzed patients.

Triple drug therapies, including an ARA and 5αRI and an MRA or mirabegron, were rare (Table 2).

Tadalafil was prescribed in 29.4% of the examined men, in 27.9% of patients on single-component preparations, and in 36.8% of patients on FDCs (*p* < 0.001) due to erectile dysfunction (59.1%), co-occurrence of erectile dysfunction and LUTS (34.9%), and rarely due to LUTS only (6.0%) in the opinion of urologists.

### 2.3. Characteristic of Subgroup on FDCs

A comparison of patients prescribed single-component drugs (SCDs) and FDCs revealed that FDCs were used more frequently in patients who were younger by 1.5 years, who were better-educated, professionally active, living in the largest towns, less symptomatic without severe LUTS, and with a prostate volume <30 mL (Table 1). In addition, the FDC group had a lower frequency of coronary artery disease, heart failure, diabetes mellitus, and recurrent urinary tract infections.

### 2.4. Changes in Pharmacotherapy Between Visits

Prior to visit 1, 15.6% and 3.9% of patients prescribed SCDs and FDCs, respectively, discontinued therapy (*p* < 0.001). At visit 1, therapy was modified in 2.5% of the continuing patients (*n* = 867), who were switched to FDCs (Figure 1).

Visit 2 took place after 2.1 ± 1.4 months. We lost 1.7% and 0.5% of patients to follow-up, while 1.3% and 2.0% of patients were prescribed single-component drugs and FDCs, respectively, excluding those switched at visit 1, discontinued therapy. Of the patients switched to FDC treatment at visit 1 (*n* = 867), 2.4% were lost to follow-up, and 6.4% discontinued treatment by visit 2 (Figure 1).

At visit 2, combination therapy based on SCDs was used by 34,341 patients (79.7% of patients, a 3.4 percentage point decrease compared to patients enrolled—83.1%), while 20.3% used FDCs (a 3.6 percentage point increase compared to patients enrolled—16.9%); see Figure 1.

No marked changes were noted in the most common ARA+5αRI combination therapies administered as SCDs and FCDs (Table 2 and Table 3) after exclusion of patients switched to FDCs at visit 1. Additionally, a decrease in tadalafil use was observed in both patients prescribed single-component drugs and fixed-dose combinations; see Table 2 and Table 3.

### 2.5. Persistence, Adherence, and Satisfaction

Persistence with therapy, assessed at visit 2, covering the period from the visit prior to inclusion, was 82.0% for SCD patients and 93.6% for FDC patients, excluding those switched at visit 1 (*p* < 0.001); see Table 4. Persistence was higher with FDCs combining tamsulosin and solifenacin than with single-component combination therapy (96.8% vs. 91.0%; *p* < 0.001). The difference in persistence between tamsulosin with dutasteride FDCs and SCD combination therapy was much smaller (85.7% vs. 80.0%; *p* < 0.001).

The 2.1-month persistence in therapy at visit 2 for patients switched to FDCs at the first visit was significantly worse than for those continuing therapy with SCDs (91.2% vs. 99.4%; *p* < 0.001)—Δ = 6.2% (95%CI: 4.4–8.3).

Better persistence with FDCs than SCDs (OR = 1.31 (95% CI: 1.02–1.63)) was independent of the patients’ characteristics, as demonstrated in a multiple regression analysis (Table 5).

Adherence was assessed at both visits. It was significantly better in patients treated with FDCs (96.6% vs. 91.0% at visit 1, *p* < 0.001; and 99.3% vs. 97.9% at visit 2, *p* < 0.05).

Those prescribed FDCs were more often satisfied or very satisfied with the therapy they received compared to those prescribed SCDs (62.6% vs. 50.6% at visit 1, *p* < 0.001; 76.8% vs. 67.5% at visit 2, *p* < 0.001). An increase in the percentage of patients who were satisfied or very satisfied with the therapy was observed in both groups. Satisfaction with FDCs was independent of the patients’ characteristics, as shown in multivariate regression analysis—OR = 1.62 (95%CI: 1.54–1.71); see Table 6.

### 2.6. Pharmacology Discontinuation and Reasons

Across two visits, 6989 patients prescribed SCDs and 500 prescribed with FDCs (17.0% vs. 5.9%; *p* < 0.001) discontinued treatment. Patient-reported dissatisfaction with effectiveness was the leading cause of treatment discontinuation for all combined therapies, followed by the cost of treatment (Table 7).

## 3. Discussion

The results of this study illustrate the spectrum of combined pharmacotherapy prescribed by urologists for LUTS associated with BPH in Poland. SCDs remain a more common form of combined therapy; however, the market share of FDCs is increasing. In this study, across a period of two visits (2.1 ± 1.4 months), the percentage of patients prescribed FDCs increased by 3.6 percentage points to 20.3%. This change was primarily caused by the use of FDCs containing tamsulosin and solifenacin. On the contrary, the increase in the frequency of using FDCs containing tamsulosin with dutasteride was markedly smaller. This indicates a habit of urologists to prescribe finasteride, which the Polish National Health Fund has reimbursed for many years. Dutasteride preparations were added to the reimbursement list in 2024. The limited availability of these medications in the form of FDCs in Poland further supports the commitment to using SCDs containing finasteride or doxazosin. We cannot exclude that the observed changes could reflect transient prescribing trends that can only be verified in subsequent studies.

As mentioned previously, FDC prescription is a widely accepted method of increasing adherence and persistence to multi-drug therapy in chronic diseases. In this analysis, the short-term adherence of patients receiving combination preparations was higher than that of patients receiving SCDs (96.2% vs. 89.7%, *p* < 0.001). It is worth emphasizing that during visit 2, the percentage of patients who complied with the recommendations increased to 97.2% (compared to 90.7% during visit 1). This can be attributed to the patients’ participation in this observational study. The persistence with therapy, assessed at visit 2, covering the period from the visit prior to inclusion, was significantly better for FDCs than SCDs by 31% (95% CI: 2–63) independently of patients’ characteristics. However, it should be stressed that the short period of follow-up precludes drawing firm conclusions concerning long-term persistence and adherence.

Combination therapies of ARAs with MRAs, like solifenacin (without 5αRI), were used less frequently (28% of combined treatments) and were administered as SCDs (containing tamsulosin or, less commonly, doxazosin) and FDCs that contained tamsulosin. The high persistence rates of 81.0% for FCD therapies based on tamsulosin with solifenacin and 70.3% for doxazosin with solifenacin are noteworthy and likely a consequence of enrolling patients who did not discontinue treatment within 3 months. As shown in the prescription database study, half the patients did not refill their MRA prescription within 30 days of the end of the initial prescription [16], with a median persistence with initiated therapy of less than 100 days [17]. Notably, in our study, persistence with tamsulosin and solifenacin FDCs was even higher at 96.8%, similar to the 95.1% persistence observed with combined therapies with mirabegron. In both cases, persistence was biased by the selection of patients who had accepted higher costs associated with the use of more expensive therapies that were not reimbursed by the Polish National Health Fund. We demonstrated that the patients prescribed FDCs were younger, better educated, professionally active, and resided in the largest towns, which supports our hypothesis of patient preselection based on awareness and a higher personal income.

In addition, we showed greater satisfaction with the use of FDCs, scored with a 5-point Visual Analog Scale. The satisfaction was greater by 62% (95%CI: 54–71), being independent of the patients’ characteristics in multivariate regression analysis. It should be noted that patient-reported dissatisfaction with the effectiveness of therapy was the main reason for discontinuation of combined therapy with FDCs and SCDs. Dissatisfaction related to the lack of improvement. A rapid improvement of LUTS, or at least stabilization of symptoms, is among the frequent patient expectations related to therapy. Others would obtain a reduction in the potential risk of surgical intervention [18].

Erectile dysfunction is a common co-occurring health problem in 71.9% of patients with BPH in Poland [15]. The frequency of this disorder in sexually active men in the study cohort reflects the frequency of tadalafil use. The study results show that three out of ten patients (29.4%) were prescribed tadalafil preparations. The indication for the use of tadalafil, provided by urologists, was specifically erectile dysfunction (59.1%) or its co-occurrence with LUTS (34.9%); rarely was it prescribed for the isolated occurrence of LUTS (6.0%). Notably, tadalafil was prescribed more frequently in patients on FDCs than in patients using SCDs (36.8% vs. 27.9%), which may be explained by younger age resulting in a higher percentage of sexually active men. It was shown that combined therapy for BPH-associated LUTS with dutasteride and high dose of solifenacin may contribute to an increase in sexual satisfaction and preservation of erectile function [19], which potentially may reduce the utilization of phosphodiesterase type 5 inhibitors, like tadalafil.

The study design had some limitations related to the collection of real-life data, which were not verified on-site by clinical monitors. Participation in this study could have encouraged doctors to use FDCs more frequently. The effect seems marginal, as the percentage of patients prescribed FDCs increased by only 3.6 percentage points during the 2.1-month study period, but even this may have distorted the described trend in the utilization of these drugs in daily clinical practice. In addition, the short follow-up period, including a 2.1-month interval between study visits and a poorly quantifiable time from the visit preceding the start of observation, precludes assessing long-term persistence with combined pharmacotherapy but allows for a comparison between SCDs and FDCs. The inclusion of a large group of patients continuing with unchanged therapy for more than 6 months (42% for FDCs and 61% for single-component preparations) could increase persistence, as the highest rates of discontinuation are expected shortly after therapy initiation [13], as confirmed in our study.

Based on the presented data, the actual frequency of combination therapy in patients with BPH managed by urologists cannot be estimated due to the non-inclusion of patients who were only actively watched and prescribed monotherapy, such as phytotherapy.

It should be emphasized that the tool used for assessing adherence was validated in patients with hypertension but not in those with BPH-associated LUTS. However, the four-item Morisky Medication Adherence Scale (MAQ) has been used in a few studies [20,21], which did not replace well-established methods for adherence evaluation, such as analyses of prescription databases or pill counting.

## 4. Materials and Methods

This multicenter, non-interventional, questionnaire-based observational study was conducted by 800 urologists and trainees managing patients with BPH in outpatient specialist care. Data concerning adherence and patient satisfaction were collected during two consecutive visits, conducted in response to the patient’s clinical needs from February to December 2024.

The inclusion criteria were male gender; age of at least 40 years; use of combination therapy for LUTS in the course of BPH, including ARAs, 5-αRIs, MRAs, and β3-adrenergic agonists, for at least 3 months before study enrollment; and verbal consent to participate. The exclusion criteria were an inability to obtain answers to the survey questions and a history of prostate surgery. Only anonymized patient data were processed.

### 4.1. Study Questionnaire

The following data were collected at visit 1: patient age, education level (primary/vocational/secondary/higher), place of residence (urban/rural), professional activity (professionally active/pensioner/retired/unemployed), marital status (single/married/widowed), and clinical data—period of time since BPH diagnosis, severity of LUTS symptoms based on IPSS, occurrence of overactive bladder symptoms (urgency, urinary incontinence, daytime frequency, and nocturia), duration of overactive bladder symptoms, prostate volume measured with sonography (30/30–40/>40 mL), last PSA test result, concomitant chronic diseases (diabetes/hypertension/coronary artery disease/heart failure/obesity/recurrent urinary tract infections/neurological diseases that may cause overactive bladder symptoms), pharmacotherapy currently used for LUTS and OAB (separate preparations or FDC)s and erectile dysfunction, duration of their use, compliance with prescription based on MAQ, patient satisfaction with pharmacotherapy used for BPH-associated LUTS (5-point Visual Analog Scale—VAS), and eventual discontinuation and reason for discontinuation (cost of treatment/occurrence of adverse events/ineffectiveness of therapy in the opinion physician/patient disappointed with the lack of effectiveness or incomplete effectiveness/disappearance of symptoms/other).

At the second visit, the collected data included the continuation of treatment or reason for its discontinuation (similar to visit 1), adherence (MAQ), current severity of LUTS (IPSS), occurrence of overactive bladder symptoms (similar to visit 1), and patient satisfaction with pharmacotherapy used for LUTS (VAS).

### 4.2. Data Analysis

The severity of lower urinary tract symptoms was classified based on the IPSS. The study group was divided into three subgroups depending on the severity of LUTS, as recommended by EAU guidelines: ≤7 points (mild), 8–19 points (moderate), ≥20 points (severe) [1].

Adherence to pharmacotherapy was assessed using the MAQ, a 4-item scale. Obtaining no more than 2 points was interpreted as adequate adherence to the medical prescription [22].

Persistence with combination therapy was scored at both visits, separately for prescribed single-component drugs and FDCs.

Patient satisfaction with pharmacotherapy for BPH-associated LUTS was analyzed based on a 5-point VAS: dissatisfied (1), slightly satisfied (2), moderately satisfied (3), satisfied (4), and very satisfied (5).

### 4.3. Statistical Analysis

Questionnaires of patients with past prostate surgery who were on monotherapy or with significant missing data were excluded from the analysis (Figure 1). No data imputation was applied. Statistical analysis was performed using MedCalc software version 20.015 (MedCalc Software Ltd., Ostend, Belgium). Qualitative data are presented as percentages, and quantitative data as means with standard deviations. Distributions of qualitative variables were compared using the χ2 test, and distributions of quantitative variables were compared using the Student’s *t* test for unpaired variables. Multivariate logistic regression models were calculated for persistence and satisfaction, including the use of FDCs, older age (≥65 years), education level (secondary or higher), urban dwelling, severe LUTS (IPSS ≥20 points), prostate volume ≥30 mL, and occurrence of OAB symptoms as predictors. A *p*-value < 0.05 was considered statistically significant.

## 5. Conclusions

1. Combination therapies are still more commonly administered as separate tablets than as FDCs in patients with BPH-associated LUTS.

2. The use of FDCs increases satisfaction and persistence with therapy, with a mild effect on adherence.

## Figures and Tables

**Table 1:** Characteristics of men with BPH-associated lower urinary symptoms on polytherapy [*n* = 50,435].

	All Subjects [*n* = 50,435]	Single-Component Drugs [*n* = 41,925]	Fixed-Dose Combination Drugs [*n* = 8510]
Age [years]	67.8 ± 8.8	68.0 ± 8.7	66.5 ± 9.1 ^
Educational level			
Elementary [*n*; %]	2861; 5.6	2298; 5.5	563; 6.6
Vocational [*n*; %]	14,467; 28.7	12,553; 29.9	1914; 22.5
Secondary [*n*; %]	18,595; 36.9	15,429; 36.8	3166; 37.2
Higher [*n*; %]	14,512; 28.8	11,645; 27.8	2867; 33.7 ^
Professional activity			
Professionally active [*n*; %]	19,071; 37.9	15,226; 36.4	3845; 45.2 ^
Pensioner [*n*; %]	30,232; 59.9	25,720; 61.3	4512; 53.0
Unemployed [*n*; %]	1132; 2.2	979; 2.3	153; 1.8
Place of residence			
Rural [*n*; %]	8080; 16.0	6904; 16.5	1176; 13.8
Town <50,000 inhabitants [*n*; %]	12,171; 24.1	10,412; 24.8	1759; 20.7
Town 50,000–100,000 inhabitants [*n*; %]	11,236; 22.3	9300; 22.2	1936; 22.7
Town > 100,000 inhabitants [*n*; %]	18,948; 37.6	15,309; 36.5	3639; 42.8 ^
Time since BPH diagnosis			
3–12 months [*n*; %]	6061; 12.0	5106; 12.2	955; 11.2
1–5 years [*n*; %]	31,881; 63.2	26,447; 63.0	5434; 63.9
6–10 years [*n*; %]	5945; 11.8	5021; 12.0	924; 10.8
>10 years [*n*; %]	6548; 13.0	5351; 12.8	1197; 14.1
Current severity of LUTS (IPSS) [pts]	13.6 ± 5.8	13.7 ± 5.9	13.2 ± 5.5
Mild [0–7 points] [*n*; %]	6874; 13.8	5660; 13.7	1214; 14.6
Moderate [8–19 points] [*n*; %]	34,315; 69.0	28,228; 68.2	6087; 73.0
Severe [20–35 points] [*n*; %]	8553; 17.2	7518; 18.1	1035; 12.4 ^
Unclassified [*n*]	693	519	174
OAB symptoms			
Urgency [*n*; %]	21,868; 43.4	18,452; 44.0	3416; 40.1 ^
Daytime frequency [*n*; %]	20,330; 40.3	17,235; 41.1	3095; 36.4 ^
Nocturnal frequency [*n*; %]	22,673; 45.0	19,182; 45.8	3491; 41.0 ^
Urinary incontinence [*n*; %]	10,674; 21.2	9586; 22.9	1088; 12.8 ^
Neurological diseases that may cause OAB [*n*; %]	2306; 4.6	1959; 4.7	347; 4.1
Prostate volume (ultrasound measurement)			
<30 mL [*n*; %]	6425; 13.5	4769; 12.0	1656; 20.5 ^
30–40 mL [*n*; %]	21,440; 45.3	18,030; 45.8	3410; 42.2
>40 mL [*n*; %]	19,552; 41.2	16,543; 42.0	3009; 37.3
Not assessed [*n*]	3018	2583	435
Last available PSA level [ng/mL]	2.3 ± 4.1	2.3 ± 4.5	1.9 ± 1.0 ^
Quality of life related to LUTS ^#^			
Excellent [0 pts]	952; 1.9	799; 1.9	153; 1.7
Good [1 point]	11,443; 23.0	9473; 22.9	1970; 23.4
Rather good [2 pts]	13,792; 27.8	11,280; 27.3	2512; 29.9
Average [3 pts]	16,238; 32.7	13,529; 32.8	2709; 32.2
Rather bad [4 pts]	5214; 10.5	4527; 11.0	687; 8.2
Bad [5 points]	1721; 3.5	1396; 3.4	325; 3.9
Very bad [6 pts]	339; 0.6	284; 0.7	55; 0.7
Missing data [*n*]	736	637	99
Comorbidity [*n*; %]	46,415; 92.0	38,794; 92.5	7621; 89.6 ^
Heart failure [A; %]	5674; 11.3	5075; 12.1	599; 7.0 ^
Coronary artery disease [*n*; %]	6935; 13.8	6120; 14.6	815; 9.6 ^
Hypertension [*n*; %]	31,352; 62.2	26,036; 62.1	5316; 62.5
Diabetes [*n*; %]	16,633; 33.0	14,326; 34.2	2307; 27.1 ^
Obesity (BMI >30.0) [*n*; %]	8816; 17.5	7254; 17.3	1562; 18.4 *
Recurrent urinary tract infection [*n*; %]	3308; 6.6	2962; 7.1	346; 4.1 ^

^#^ How would you feel if your urinary symptoms continued at their current level? Statistical significance vs. single-component drugs: * *p* < 0.05; ^ *p* < 0.001. BPH—benign prostatic hyperplasia; IPSS—International Prostate Symptom Score; LUTS—lower urinary tract symptoms; OAB—overactive bladder.

**Table 2:** Characteristics of combination pharmacotherapies used to treat BPH-associated lower urinary tract symptoms at visit 1 in the study group.

	Single-Component Drugs [*n* = 41,925]	Fixed-Dose Combination Drugs [*n* = 8510]	*p*
ARA with 5αRI	32,937; 78.6	2465; 29.0	<0.001
Tamsulosin + Finasteride [*n*; %]	24,369; 58.1	-	-
Tamsulosin + Dutasteride [*n*; %]	4827; 11.5	2465; 29.0	<0.001
Doxazosin + Finasteride [*n*; %]	2834; 6.8	-	-
Doxazosin + Dutasteride [*n*; %]	907; 2.2	-	-
ARA with MRA or β3-adrenergic agonist	8853; 21.1	6045; 71.0	<0.001
Tamsulosin + Solifenacin [*n*; %]	6632; 15.8	6045; 71.0	<0.001
Doxazosin + Solifenacin [*n*; %]	1398; 3.3	-	-
Tamsulosin/Doxazosin + Mirabegron [*n*; %]	823; 2.0	-	-
5αRI with MRA	56; 0.1	-	-
Dutasteride/Finasteride + Silodosin [*n*; %]	56; 0.1	-	-
ARA with 5αRI with MRA or β3-adrenergic agonist	79; 0.2	-	-
Tamsulosin + Finasteride + Solifenacin [*n*; %]	79; 0.2	-	-
Duration of therapy with the current drug			
≤6 months [*n*; %]	16,339; 39.0	4938; 58.0	<0.001
7–12 months [*n*; %]	5937; 14.1	1235; 14.5	0.40
>12 months [*n*; %]	19,649; 46.9	2337; 27.5	<0.001
Adherence (MAQ ≤ 2 pts) [*n*; %]	37,452; 91.0	7872; 96.6	<0.001
Missing data [*n*]	783	359	-
Patient satisfaction with pharmacotherapy for LUTS			
Dissatisfied [*n*; %]	1136; 2.7	65; 0.8	<0.001
Slightly satisfied [*n*; %]	3048; 7.3	442; 5.2	<0.001
Moderately satisfied [*n*; %]	16,381; 39.4	2666; 31.4	<0.001
Satisfied [*n*; %]	19,743; 47.5	4824; 56.7	<0.001
Very satisfied [*n*; %]	1290; 3.1	502; 5.9	<0.001
Missing data [*n*]	327	11	-
Tadalafil [*n*; %]	11,687; 27.9	3130; 36.8	<0.001

5-αRI—5-α reductase inhibitor; ARA—α1-adrenergic receptor antagonist; LUTS—lower urinary tract symptoms; MAQ—Morisky Medication Adherence Scale; MRA—muscarinic receptor antagonist.

**Table 3:** The pharmacotherapy of BPH-associated lower urinary tract symptoms, adherence, and patient satisfaction at the second visit.

	Single-Component Drugs [*n* = 34,341]	Fixed-Dose Combination Drugs [*n* = 7966] *	*p*
ARA with 5αRI [*n*; %]	27,139; 92.8	2113; 7.2	
Tamsulosin + Finasteride [*n*; %]	20,706; 60.4	-	-
Tamsulosin + Dutasteride [*n*; %]	3860; 11.2	2113; 26.5	<0.001
Doxazosin + Finasteride [*n*; %]	2111; 6.1	-	-
Doxazosin + Dutasteride [*n*; %]	462; 1.3	-	-
ARA with MRA or β3-adrenergic agonist [*n*; %]	7135; 54.9	5853; 45.1	
Tamsulosin + Solifenacin [*n*; %]	5369; 15.6	5853; 73.5	<0.001
Doxazosin + Solifenacin [*n*; %]	983; 2.9	-	-
Tamsulosin/Doxazosin + Mirabegron [*n*; %]	783; 2.3	-	-
5αRI with MRA [*n*]	31	-	
Dutasteride/Finasteride + Silodosin [*n*; %]	31; 0.1	-	-
ARA with 5αRI with MRA or β3-adrenergic agonist [*n*]	36	-	
Tamsulosin + Finasteride + Solifenacin [*n*; %]	36; 0.1	-	-
Adherence (MAQ ≤ 2 pts) [*n*; %]	32,978; 97.9	7466; 99.3	<0.001
Missing data [*n*]	642	446	-
Patient satisfaction with pharmacotherapy for LUTS [pts]	3.7 ± 0.9	3.9 ± 0.9	<0.001
Dissatisfied [1 pts] [*n*; %]	65; 0.2	11; 0.1	
Slightly satisfied [2 pts] [*n*; %]	1052; 3.0	185; 2.3	
Moderately satisfied [3 pts] [*n*; %]	10,069; 29.3	1653; 20.8	<0.001
Satisfied [4 pts] [*n*; %]	19,906; 58.0	4727; 59.3	
Very satisfied [5 pts] [*n*; %]	3249; 9.5	1390; 17.5	
Tadalafil [*n*; %]	5317; 15.5	1846; 23.2	<0.001

* For patients not switched to fixed-dose combination drugs at visit 1. 5-αRI—5-α reductase inhibitor; ARA—α1-adrenergic receptor antagonist; LUTS—lower urinary tract symptoms; MAQ—Morisky Medication Adherence Scale; MRA—muscarinic receptor antagonist.

**Table 4:** Discontinuation and persistence with therapy across two visits for specific combination therapies. Small subgroups (<100 patients) were excluded from the analysis.

	Single-Component Drugs		Fixed-Dose Combination Drugs	
	Discontinuation [*n*; %]	Persistence [*n*; %]	Discontinuation [*n*; %]	Persistence [*n*; %]
[*n*; %]	7521; 18.0	34,274; 82.0	544; 6.4	7966; 93.6 ^
ARA with 5αRI	5803; 17.6	27,139; 82.4		
Tamsulosin + Finasteride	3663; 15.0	20,706; 85.0		
Tamsulosin + Dutasteride	967; 20.0	3860; 80.0	352; 14.3	2113; 85.7 ^
Doxazosin + Finasteride	723;25.5	2111; 74.5		
Doxazosin + Dutasteride	445; 49.1	462; 50.9		
ARA with MRA or β3-adrenergic agonist	1718; 19.4	7135; 80.6		
Tamsulosin + Solifenacin	1263; 19.0	5369; 81.0	192; 3.2	5853; 96.8 ^
Doxazosin + Solifenacin	415; 29.7	983; 70.3		
Tamsulosin/Doxazosin + Mirabegron	40; 4.9	783; 95.1		

^ *p* < 0.001 vs. single-component therapy. 5-αRI—5-α reductase inhibitor; ARA—α1-adrenergic receptor antagonist; MRA—muscarinic receptor antagonist.

**Table 5:** Factors affecting persistence with combination BPH-related LUTS therapy across two visits. Results of multivariate regression analysis.

Variable	Level	OR (95%CI)
Pharmacotherapy	SDCs	Ref
	FDCs	1.31 (1.02–1.63) *
Age	<65 yrs	ref
	≥65 yrs	0.77 (0.66–0.90) **
Education level	Elementary/Vocational	Ref
	Secondary/Higher	1.55 (1.34–1.79) ***
Place of residence	City	Ref
	Rural	0.65 (0.52–0.80) ***
LUTS	0–19 pts	Ref
	20–35 pts	1.83 (1.25–2.68) **
Prostate volume	<30 mL	Ref
	≥30 mL	0.89 (0.77–1.02)
OAB	No	Ref
	Yes	1.40 (1.11–1.76) **

* *p* < 0.05; ** *p* < 0.01; *** *p* < 0.001; FDCs—fixed-dose combination medication; LUTS—lower urinary tract symptoms; OAB—overactive bladder; SCD—single-component drug.

**Table 6:** Factors affecting satisfaction with therapy across two visits. Results of multivariate regression analysis.

Variable	Level	OR (95%CI)
Pharmacotherapy	SDCs	Ref
	FDCs	1.62 (1.54–1.71) ***
Age	<65 yrs	ref
	≥65 yrs	0.97 (0.93–1.02)
Education level	Elementary/Vocational	Ref
	Secondary/Higher	1.23 (1.17–1.28) **
Place of residence	City	Ref
	Rural	1.56 (1.47–1.64) ***
LUTS	0–19 pts	Ref
	20–35 pts	0.11 (0.10–0.12) ***
Prostate volume	<30 mL	Ref
	≥30 mL	0.55 (0.53–0.57) ***
OAB	No	Ref
	Yes	0.12 (0.11–0.14) ***

** *p* < 0.01; *** *p* < 0.001; FDC—fixed-dose combination medication; LUTS—lower urinary tract symptoms; OAB—overactive bladder; SCDs—single-component drugs.

**Table 7:** Pharmacotherapy discontinuation and reasons across two visits.

Fixed-dose combined drugs [*n*]	500
Tamsulosin with dutasteride [*n*]	257
Patient-reported dissatisfaction with effectiveness [*n*; %]	154; 59.9
Cost of treatment [*n*; %]	41; 16.0
Missing data [*n*; %]	62; 24.1
Tamsulosin with solifenacin [*n*]	243
Patient-reported dissatisfaction with effectiveness [*n*; %]	177; 72.8
Cost of treatment [*n*; %]	32; 13.2
Missing data [*n*; %]	34; 14.0
**Single-component drugs [*n*]**	**6989**
Tamsulosin or Doxazosin with Finasteride or Dutasteride [*n*]	4969
Patient-reported dissatisfaction with the effectiveness [*n*; %]	3873; 77.9
Cost of treatment [*n*; %]	243; 4.9
Missing data [*n*; %]	853; 17.2
Tamsulosin or doxazosin with solifenacin [*n*]	1553
Patient-reported dissatisfaction with effectiveness [*n*; %]	1089; 70.2
Cost of treatment [*n*; %]	229; 14.7
Missing data [*n*; %]	235; 15.1

## Data Availability

The datasets are available from the Europharma Research and Science Centre Co., Ltd. upon reasonable request (agnieszka@europharma.edu.pl).

## Abbreviations

The following abbreviations are used in this manuscript:

FDCs	Fixed-dose combination medications
SCDs	Single-component drugs
BPH	Benign prostatic hyperplasia
LUTS	Lower urinary tract symptoms
ARAs	α1-adrenergic receptor antagonists
EAU	European Association of Urology
5-αRIs	5-α reductase inhibitors
MRAs	Muscarinic receptor antagonists
IPSS	International Prostate Symptom Score
AUASI	American Urological Association Symptom Index
AUR	Acute urinary retention
TURP	Transurethral Resection of the Prostate
OAB	Overactive bladder
VAS	Visual Analog Scale
MAQ	Morisky Medication Adherence Scale
